# Editorial: Beyond traditional antibiotics: innovations in biologics, chemical modulators, and microbiome manipulation

**DOI:** 10.3389/fphar.2025.1679268

**Published:** 2025-09-10

**Authors:** Anutthaman Parthasarathy

**Affiliations:** University of Bradford, Bradford, United Kingdom

**Keywords:** antimicrobial resistance (AMR), antimicrobial peptides (AMP), anti-infective bone cement, mAb-monoclonal antibody, probiotic

The worldwide trend of increasing antimicrobial resistance has prompted research into a variety of alternative strategies to fight microbial infections such as the use of bacteriophages, engineered antibodies, antimicrobial peptides, chemical modulation, microbiome manipulation and novel technologies. These form the topic of this Research Topic. Our present Research Topic covers repurposed monoclonal antibodies (mABs), antimicrobial peptides (AMPs), anti-infective bone cements, a probiotic fungus and antibiotic-free infection management.

Modulating the host immune system is a way to reduce antimicrobial drug usage, and Gao et al. explore the use of a repurposed monoclonal antibody tocilizumab (which targets the interleukin–6 receptor (IL–6R) and was approved for rheumatologic conditions), to treat critically ill COVID patients in a single-centre study. While all patients who took 1–3 doses of this drug showed improved levels of inflammatory markers, mortality or secondary complications did not significantly change with increasing doses.

In the search for novel antimicrobials, AMPs are a leading natural product class with multi-mechanism antimicrobial effects. Among AMPs, bacteriocins are produced mainly by *Bacillus* strains largely to inhibit closely related bacteria. Li et al. report the characterisation of *Bacillus velezensis* bacteriocin P7 active against *Staphylococcus aureus*. Since media composition has a great effect on bacteriocin production, the media optimisation efforts are also reported.

Liu et al. review a special type of antimicrobial material, namely calcium phosphate based anti-infective bone cements. Bone infections (often caused by *S. aureus*) are hard to treat with conventional antibiotics due to the nature of bone material. The high systemic antibiotic concentrations needed poses a considerable risk of AMR. Calcium phosphate cements (CPCs) are bone-compatible materials which can include anti-infective agents such as antibiotics, AMPs, graphene and antibacterial inorganic ions classified under therapeutic inorganic ions (TIIs).

Li and Xie present a meta-analysis of the probiotic *Saccharomyces boulardii* as a microbiome manipulator to manage *H. pylori* infections. They review 19 studies and conclude that when *S. boulardii* is supplemented with conventional treatments, *H. pylori* eradication works significantly better. Furthermore, the disease symptoms such as bloating, nausea, diarrhoea and constipation are reduced.

Shariati et al. review a range of antibiotic-free strategies to manage Carbapenem-resistant (CR) Gram-negative bacteria, whose infections present significant risks of mortality. These include not only bacteriophages and AMPs but also small-molecule natural products, nanoparticles, the modified amino acid N-acetylcysteine and the approved anti-addiction drug disulfiram.

Parthasarathy et al. report that a powerful antibiotic cocktail active against multidrug-resistant MDR pathogens is produced by the interaction of co-isolated Gram-positive *Exiguobacterium* and Gram-negative *Acinetobacter*, and that the lead compounds derived from chromatography of the crude extract include several AMPs.

In summary, this Research Topic offers a snapshot of some important evolving modalities to limit the evolution of AMR. This includes the use of repurposed mABs, repurposed small molecule drugs, AMPs, bacteriophages, the combination of probiotics with existing antibiotic-based anti-infective therapeutics, and anti-infective bone cements which can be loaded with antibiotics or antibiotic alternatives.

